# Networks as a Type of Social Entrepreneurship to Advance Population Health

**Published:** 2010-10-15

**Authors:** Jane Wei-Skillern

**Affiliations:** Haas School of Business, University of California, Berkeley

## Abstract

A detailed case study from the field of social entrepreneurship is used to illustrate the network approach, which does not require more resources but rather makes better use of existing resources. Leaders in public health can use networks to overcome some of the barriers that inhibit the widespread adoption of a population health approach to community health. Public health leaders who embrace social entrepreneurship may be better able to accomplish their missions by building their networks rather than just their organizations.

## Social Entrepreneurship and Networks

Social entrepreneurship has become prominent as an approach to address societal problems. The term is generally conceptualized as innovative activity within or across the nonprofit, government, or business sectors to generate social impact (eg, improvements in public health, environmental conservation, economic development) (1). As traditional approaches to addressing society's ills have failed, social entrepreneurship is seen as a way to leverage resources, enhance effectiveness through innovative partnerships, raise levels of performance and accountability, and ultimately achieve sustainable social impact.

Social entrepreneurship builds on the definition of entrepreneurship as "the pursuit of opportunity beyond the resources that you currently control" (2). Conceptualizations of social entrepreneurship (3) are based on the drive to create social impact rather than personal or shareholder wealth. Social entrepreneurship is often characterized by some of the virtues of commercial entrepreneurship, such as efficiency, dynamism, innovativeness, high performance, and economic sustainability. Examples of such social entrepreneurship include nonprofits operating revenue-generating enterprises (4-6) or pursuing organizational growth (7) to increase the quantity or quality of programs or services. Undoubtedly, many social-sector organizations, following in the footsteps of their commercial counterparts, have achieved substantial impact by attracting more resources, developing their organizational infrastructure, and increasing the scale of their operations. Yet, the process of organizational growth also poses tremendous challenges, particularly in the social sector (those organizations whose primary goal is serving the public interest) where human and financial capital is often scarce. Even organizations that overcome obstacles to growth and achieve appreciable scale seldom achieve substantial social impact on their own.

Some researchers and practitioners have argued that the opportunities and challenges in the social sector require not only the creative use of commercial approaches but also the development of new conceptual frameworks and strategies tailored specifically to generating social impact. A prime example of this conceptualization of social entrepreneurship is a network approach. In a network approach, leaders not only focus on management challenges and opportunities at an organizational or institutional level but also try to mobilize resources more broadly within and outside traditional boundaries to generate maximum social impact.

Although social impact can be generated through traditional means by bringing resources into an organization and delivering programs or services directly, organizations can often achieve greater social impact by leveraging the resources and expertise of complementary, or even competing, organizations. By forming networks, leaders can mobilize resources and activities across unit, organizational, and sector boundaries to achieve maximum social impact. I conclude by describing how networks can be used by leaders in public health to overcome some of the barriers to adoption of a population health approach to community health.

## A Network Case Study

Organizations that have consistently achieved and sustained substantial social impact despite limited resources have done so by working through networks (8-12). The example of the Guide Dogs for the Blind Association (GDBA) illustrates some of the factors that are important to successful network building (13).

GDBA, a charity based in the United Kingdom, is the world's largest breeder and trainer of guide dogs. In 1997, the chief executive officer, Geraldine Peacock, realized that the public sector that was supposed to deliver services to visually impaired people was not working efficiently or effectively. GDBA was providing guide dogs to just 5,000 clients, despite its 66-year history and considerable organizational scale: an annual budget of approximately 40 million pounds (US $58.5 million), 27 offices across the United Kingdom, and a staff of approximately 1,200. The organization's own research found that in the United Kingdom approximately 200,000 people needed mobility services, including not only guide dogs but also other services, such as long cane mobility training. At the same time, the organization was losing millions of pounds per year because it had expanded its programs into noncore areas such as operating hotels for the visually impaired.

Peacock sought to improve the organization's effectiveness in several ways. First, she divested GDBA of operations that were not core to GDBA's mission, such as the hotels program. She engaged trusted partners who would have the capacity to take ownership of the divested operations and invested millions of pounds in these partners to ensure their partners' success in running those programs. Second, to improve services overall, GDBA partnered with local governments, which had responsibility for providing services such as mobility training, independent living skills, and communication skills. GDBA offered to pay for the mobility training that was the responsibility of the government, because the mobility training programs were chronically underfunded and mobility training was GDBA's core expertise. The government could have GDBA provide mobility training directly or could use the funds from GDBA to hire a local nonprofit provider. In the latter case, GDBA also offered to provide technical assistance to support its former "competitors" in providing services to visually impaired people. According to Peacock, it was less important who provided the services than whether they were being provided at a high quality. In exchange for GDBA's resources, the government contractually committed to match 1:1 the funds that GDBA provided for mobility training and use them for independent living and communication skills services. Peacock deliberately pursued a strategy that supported building capacity in the field and facilitating collaborations among providers that had historically been competitive with each other.

Finally, Peacock sought to enhance the efficiency and effectiveness of the charities serving the visually impaired by creating an umbrella organization that would offer a unified voice and a shared advocacy agenda. The individual organizations maintained their own brands and operations, but the umbrella facilitated more frequent communication and ongoing collaborations among organizations in the field.

Within 5 years of creating these partnerships, GDBA more than doubled the number of clients who received mobility training without increasing its own operations. After witnessing the success of GDBA's network approach, in 2002 the UK government established a fund of 125 million pounds (US $182.5 million) to invest in the types of networks that GDBA and its partners had pioneered.

At GDBA and other organizations using this approach, common factors for effective networks emerge. These networks depend on a willingness among all participants to shift their focus from maximizing organizational- and institutional-level benefits to maximizing social impact. Thus, network participants must be willing to 1) invest substantial resources (financial being just one), 2) share or relinquish control, and 3) share rewards and recognition with their partners. The network approach also benefits organizations that use it. The network approach enabled GDBA, for example, to change its own culture and reputation from that of an independent, and at times domineering, organization to one that government and other nonprofits consider a trusted partner.

## The Need for Social Entrepreneurship in Population Health

Although the term *social entrepreneurship* has emerged recently in the field of public health, the concept itself is nothing new in public health practice. Partnerships are becoming more common between the medical and public health communities to coordinate vaccination, case reporting, and education on such issues as childhood diseases and sexually transmitted diseases, among others. In addition, a joint medical and public health professional association was created (14). The notion that involvement of communities is necessary for developing effective and sustainable public health interventions has become widely accepted (15,16). Research has documented the effectiveness of approaches that draw on local, national, and global knowledge-sharing and support across issues such as reducing cesarean rates, hospital delays and wait times, and hospital admissions for asthma (17,18). Research on patient safety has documented the importance of system-level approaches to improving population health (19).

The emergence of the field of population health, which emphasizes a holistic and system-level understanding of "health outcomes, patterns of health determinants, and policies and interventions that link these two" (20), tempers the rising dominance of the perception that health care is the primary determinant of health outcomes. Many other nonmedical determinants, such as the social and physical environment, individual behavior, and genetics, are factors in population health (20). Just as pay-for-performance might improve the quality of medical care, similar pay-for-population health performance systems should be developed. Financial and nonfinancial incentives are a positive and necessary step to motivate system-level thinking and action toward population health goals. However, achieving the objectives of any pay-for-population health system also requires a fundamental change in the culture and mindset of the leaders and actors in the health fields, both medical and nonmedical. As illustrated in the GDBA example, leaders must let go of traditional notions of their organizations and agencies as hubs and potential partners as mere spokes. Instead, leaders must view their organizations and their work as nodes among many others in a larger constellation of actors that must coordinate their efforts to achieve a shared vision. To lead their organizations to greater efficiency, effectiveness, and sustainability, they need to creatively mobilize resources beyond their control in the name of improved population health outcomes. The work of any single agency or organization, while important, can contribute in substantial ways to population health improvements only to the extent that it is linked and supported by other system-level efforts.

The sector of population health shares many of the characteristics of other social sectors, which makes it amenable to social entrepreneurship and, specifically, to network approaches:

- Organizations seek to address large, complex issues that cannot be addressed by any single entity.
- Organizations seek to create social impact, not just organizational impact.
- Organizations often have dispersed governance and accountability.
- Organizations create value that is not readily measured.
- Organizations rely heavily on tacit knowledge and expertise as well as trust and relationships to achieve social impact.

Although large-scale health challenges require solutions that no single agency or institution can tackle, virtually all incentive systems in public health preclude such system-level solutions. Funders, governing boards, donors, and organizational and institutional leaders often seek organizational growth and revenue increases rather than impact as primary goals. Board members of various public health agencies are accountable only for their organizations, not how effectively their organization's work is integrated with the system on which population health outcomes depend. Many donors encourage collaboration among grantees, but they often assume that because they bring the financial resources they can also dictate solutions when in fact the keys to solving the problem are dispersed across individuals and entities throughout the community. Furthermore, donors often restrict funding to specific programs rather than granting discretion to the grantees. Dictating programs and how they should be delivered severely limits the creativity and flexibility that local experts and leaders need to build network solutions. Given this state of affairs, one would not expect health care and health institution leaders to be focused on anything but their own organization's well-being. Yet, recent research in the field of social entrepreneurship suggests that a network mindset (21) may offer a promising tool to overcome the barriers to achieving population health.

## Applying Networks to Overcome Barriers to Pay-for-Population Health

Networked organizations are different from traditional organizations in that they look outward rather than inward. They put their vision and mission first and their organizations second. They govern through trust rather than top-down controls. They cooperate as equal nodes in a broad network of actors rather than strive to become a central hub that dictates the agenda. A shift from the organizational to the networked mindset offers solutions to some of the barriers to pay-for-population health systems identified by public health experts (20):

1. **No consensus on how to measure population health.** The network approach suggests that it may not be necessary for the field of population health to come to consensus on a single metric at the outset. The goal is to get leaders in the field to focus on population health outcomes, allowing flexibility around what the outcomes might be and the means for achieving them. As self-organizing clusters of networks around shared metrics begin to emerge, the actors themselves may begin to gravitate toward the metrics that have the greatest merit.
2. **Financial incentives and unintended consequences.** Financial incentives should reward organizations that show an enduring commitment to population health goals through their actions. Trust is fundamental to enabling networks to thrive. If participants fear that they will be exploited by their network partners, the focus reverts to self-interest. Effective network builders seek out peers with similar values to build systemic solutions; ineffective network participants will remain isolated at the margins. Funders can reward the former and limit funding for the latter.
3. **Coordination across sectors.** A network approach introduces a shift in thinking about coordination not only by breaking down silos through vertical integration but also by investing heavily to foster the development of lateral relationships among various organizations and sectors. Donors might host meetings, provide venues for health care and public health leaders and providers to discuss specific population health issues, and offer resources to support innovative forms of collaboration. This approach is particularly promising because it does not require cumbersome large-scale acquisitions or mergers. Coordination can start small in multiple arenas and expand as the partners build trust and see the fruits of their partnership. As organizations experience the mutual benefits of collaboration, they may also identify more substantive areas of work. For example, they may mobilize around a holistic approach to disease treatment and management, such as for diabetes, through which patients could benefit substantially from coordinated interventions, such as nutrition, exercise, and medical care. Not all partnerships are destined to flourish, and not all partners are trustworthy, but facilitating peer-to-peer relationship-building and cooperation may catalyze relationships that ultimately contribute to better population health.
4. **Resistance to reallocation of resources.** Leaders must realize that maximizing their own organizational resources is not a true measure of success; instead, health outcomes should be the measure. More efficiency can be achieved through collaboration, thereby reducing costs and attracting more funding from donors that go out of their way to fund effective network builders rather than organization builders.
5. **Focus on current issues rather than preventing tomorrow's population health problems.** Any pay-for-population health system must seek to reward leaders and organizations that build networks to deliver system-level solutions rather than investing in their own sustainability. Few leaders seek to drive their organizations out of business, yet in the social sector, that is precisely what the goal should be. Career paths that span the field and sector must be developed to replace career paths tied to specific organizations.

Although no silver bullet can magically answer the population health challenge, a social entrepreneurial approach using networks expands the horizon for innovative solutions. The network approach is particularly powerful because it does not require more resources but instead makes better use of existing resources.
